# Nicotine Causes Mitochondrial Dynamics Imbalance and Apoptosis Through ROS Mediated Mitophagy Impairment in Cardiomyocytes

**DOI:** 10.3389/fphys.2021.650055

**Published:** 2021-06-10

**Authors:** Ting-ting Meng, Wei Wang, Fan-liang Meng, Shu-ya Wang, Hui-hui Wu, Jia-min Chen, Yan Zheng, Guang-xin Wang, Mao-xiu Zhang, Ying Li, Guo-hai Su

**Affiliations:** ^1^Research Center of Translational Medicine, Jinan Central Hospital, Cheeloo College of Medicine, Shandong University, Jinan, China; ^2^Research Center of Translational Medicine, Central Hospital Affiliated to Shandong First Medical University, Jinan, China; ^3^Department of Cardiology, Shandong Provincial Chest Hospital, Jinan, China; ^4^The Affiliated Hospital of Shandong University of Traditional Chinese Medicine, Jinan, China

**Keywords:** nicotine, mitophagy, mitochondria dynamics, apoptosis, CSTL

## Abstract

Nicotine contained in traditional cigarettes, hookahs, and e-cigarettes is an important risk factor for cardiovascular disease. Our previous study showed that macroautophagic flux impairment occurred under nicotine stimulation. However, whether nicotine influences mitochondrial dynamics in neonatal rat ventricular myocytes (NRVMs) is unclear. The purpose of this study was to explore the effects and potential mechanism of nicotine on mitophagy, mitochondrial dynamics, apoptosis, and the relationship between these processes in NRVMs. Our results showed that nicotine exposure increased mitochondria-derived superoxide production, decreased mitochondrial membrane potential, and impaired PINK1/Parkin-mediated mitophagic flux in NRVMs. Interestingly, nicotine significantly promoted dynamin-related protein 1 (Drp1)-mediated mitochondrial fission and suppressed mitofusin (MFN)-mediated fusion, which was also observed in the bafilomycin A1-treated group. These results suggest that mitophagic flux impairment may contribute to Drp-1-mediated mitochondrial fission. Finally, nicotine caused excessive mitochondrial fission and contributed to apoptosis, which could be alleviated by mdivi-1, an inhibitor of Drp1. In addition to CTSB, as we previously reported, the enzyme activity of cathepsin L (CTSL) was also decreased in lysosomes after stimulation with nicotine, which may be the main cause of the hindered mitophagic flux induced by nicotine in NRVMs. Pretreatment with Torin 1, which is an inhibitor of mTOR, activated CTSL and ameliorated nicotine-induced mTOR activation and mitophagy impairment, decreased mitochondria-derived superoxide production, and blunted mitochondrial fission and apoptosis. Pretreatment with the ROS scavenger N-acetyl-cysteine (NAC) or inhibitors of p38 and JNK, which could also alleviate mitophagy impairment, exhibited similar effects as Torin1 on mitochondria. Taken together, our study demonstrated that nicotine treatment may lead to an increase in Drp1-mediated mitochondrial fission by blocking mitophagic flux by weakening the enzyme activity of CTSL and activating the ROS/p38/JNK signaling pathway. Excessive mitochondrial fission induced by nicotine ultimately leads to apoptosis. Torin1 restored the decreased CTSL enzyme activity by removing excessive ROS and alleviated the effects of nicotine on mitophagic flux, mitochondrial dynamics, and apoptosis. These results may provide new evidence on the relationship between mitophagic flux and mitochondrial dynamics and new perspectives on nicotine’s effects on mitochondrial dynamics in cardiomyocytes.

## Introduction

As the main component of tobacco, nicotine is associated with an increased risk of cardiovascular diseases (Benowitz and Fraiman, 2017; Darville and Hahn, 2019). It can not only promote hypertension-related vascular endothelial dysfunction, but also promote oxidative stress, inflammation, fibrosis and apoptosis, and affect the structure and function of the heart (Benowitz and Burbank, 2016). However, the potential mechanism of nicotine-induced cardiomyocyte injury is not clear.

Mitochondria may be a potential target of nicotine, and an organelle plays a central role in the regulation of cardiac function and cardiomyocyte survival (Fuhrmann and Brüne, 2017; Kozlov et al., 2017; Malińska et al., 2019). In response to environmental changes, mitochondria can quickly change from a source of energy for fueling contractile function to a trigger of cell death. Mitochondria can not only function as producers of excessive reactive oxygen species (ROS) and factors that participate in cell death pathways but are also particularly susceptible to oxidative injury (Kang et al., 2020). Thus, mitochondrial dysfunction induced by ROS could further enhance the production of ROS, leading to a vicious feed-forward cycle, which causes sustained oxidative damage (Suzuki et al., 2015). Mitophagy is a defense mechanism developed against aberrant mitochondria in cells and is important in maintaining the quality of mitochondria. It is a cellular process that selectively removes aging, pathological or damaged mitochondria via the specific sequestration and engulfment of mitochondria for subsequent lysosomal degradation (Ma et al., 2020). Our previous study showed that nicotine could cause cellular ROS accumulation and induce autophagic flux impairment (Wang et al., 2020). However, the effects and the mechanism of nicotine on mitophagy still need further investigated.

Mitochondrial dynamics, which are referred to as mitochondrial fission and fusion, play an important role in shaping and distributing mitochondria and contributing to mitochondrial homeostasis and adaptation to stress (Terriente-Felix et al., 2020). Destruction of the equilibrium of mitochondrial dynamics is implicated in various diseases, including cancer (Srinivasan et al., 2017), neuron degeneration (Ishikawa et al., 2020), type 2 diabetes (Rovira-Llopis et al., 2017; Williams and Caino, 2018) and osteoarthritis (Yao et al., 2019; He et al., 2020). Mitochondrial fusion is considered to be beneficial since it is associated with an increase in mitochondrial function and ATP production. In contrast, excessive mitochondrial fission seems to be detrimental since it is associated with decreased mitochondrial function and increased ROS (Ding et al., 2018). However, whether nicotine is involved in the regulation of mitochondrial dynamics and the potential mechanism in NRVMs is unknown.

Recently, mitophagy was proven to be closely related to mitochondrial dynamics (Xian and Liou, 2020). Short mitochondria induced by mitochondrial fission can be easily targeted and eliminated by mitophagy (Burman et al., 2017; Chen et al., 2016; Twig et al., 2008; Chen and Dorn, 2013). Mitochondrial fission is crucial to the initiation of mitophagy, and mitochondrial fusion plays the role of a savior preventing mitophagy (Twig et al., 2008; Twig and Shirihai, 2011; Youle and van der Bliek, 2012). On the other hand, mitochondrial fusion maintains the quality of mitochondria by diluting the dysfunctional components of mitochondria, which inhibits the progression of mitophagy to some extent (Xian and Liou, 2020). However, the relationship between mitophagy and mitochondrial dynamics is still controversial (Burman et al., 2017; Murakawa et al., 2015; Chen et al., 2017; Xian and Liou, 2020). The effects of hindered mitophagic flux on mitochondrial dynamics are largely unknown.

The purpose of the present study was to investigate the effects of nicotine on mitophagy, mitochondrial dynamics and apoptosis and the relationship between these processes in neonatal rat ventricular myocytes (NRVMs) and the potential mechanism. Our results showed that nicotine promoted Drp1-mediated mitochondrial fission by hindering mitophagic flux by weakening the enzyme activity of CTSL and activating the ROS/p38/JNK signaling pathway. These results may provide new proof of a close relationship between mitophagic flux and mitochondrial dynamics, which is also the first demonstration focused on nicotine’s effects on mitochondrial dynamics in NRVMs.

## Materials and Methods

### Primary Rat Cardiomyocytes Culture and Adenovirus Transfection

The primary cardiomyocytes of rats were cultured as previously described (Li et al., 2016). Briefly, after 1- to 3-day-old Wistar rats were anesthetized with isoflurane, the ventricles were digested with Hanks’ solution containing 200 U of collagenase and 0.4% horse serum. The digested cells were then centrifuged and cultured in Dulbecco’s modified Eagle’s medium containing 5% fetal bovine serum and 8% horse serum. Differential adhesion (1.5 h) was used to purify cardiomyocytes. The purified cells were proven by morphological examination and staining with an anti-sarcomeric-actin antibody. Then, 0.1 mM 5-bromo-2-deoxyuridine (BrdU) was added to primary cardiomyocytes for 3 days. GFP-RFP-Fis1 (2 × 10^10 PFU/ml in stock solution, 1:5000-1:200000 dilution) was used to infect NRVMs for 24 h and then proceeded to the next step of the experiment (Allen et al., 2013). The animal care and experimental procedures involved in this study were reviewed and approved by Ethics Committee of Jinan Central Hospital (approval code: AF/SC-07/02.0).

### Reagents

SP600125, SB203580, and N-acetyl cysteine (NAC) were obtained from Sigma-Aldrich. Torin1 and Mdivi-1 were purchased from Selleck. Antibodies against mitofusin (MFN)-1, mitofusin (MFN)-2, PARP, caspase3, p62, and β-actin were obtained from Proteintech. Antibodies against dynamin-related protein (Drp)-1 were obtained from ABclonal. Antibodies against cleaved caspase-3, cleaved PARP, and LC3 were obtained from Cell Signaling Technology.

### Real-Time Polymerase Chain Reaction

TRIzol reagent was used to isolate RNA from NRVMs. Reverse transcriptions (RTs), and real-time qPCRs were performed as previously described (Wang et al., 2020). Primers used in this study were as follows: Dnm1l, Forward primer 5′-TGGAAAG AGCTCAGTGCTGG-3′, Reverse primer 5′-ACTCCATTTT CTTCTCCTGTTGT-3′; MFN1, Forward primer 5′-TGACT TGGACTACTCGTGCG-3′, Reverse primer 5′-GTGGCCATT TCTTGCTGGAC-3′; MFN2, Forward primer 5′-GTTCAGAGG CCATCGGTTCA-3′, Reverse primer 5′-GTGCTTGAGAGG GGAAGCAT-3′; β-actin, Forward primer 5′-CGTTGACATC CGTAAAGACC-3′, Reverse primer 5′-TAGAGCCACC AATCCACACA-3′. LightCycler 480II Fast RealTime PCR System (Roche, Switzerland) was used to quantify the level of relative mRNA, which was normalized to β-actin sequentially.

### Western Blotting Analysis

Western blotting was performed as previously described (Wang et al., 2020). In brief, protein was extracted from NRVMs by cell lysis buffer, and 5x loading buffer was added to stabilize the structure. Sodium dodecyl sulfate-polyacrylamide gel electrophoresis (SDS-PAGE) was used to separate proteins with different molecular weights. Processed proteins were transferred to a PVDF membrane and incubated with various antibodies, such as anti-cleaved-caspase3, anti-caspase3, anti-cleaved-PARP, anti-PARP, anti-PINK1, anti-Parkin, anti-MFN1, anti-MFN2, anti-Drp1, and anti-β-actin antibodies (1:1,000 dilution). Related proteins were detected against rabbit or mouse IgG conjugated to horseradish peroxidase. Quantity One software (Bio-Rad, United States) was used to quantify proteins.

### Mitochondrial ROS Detection

Mitochondrial ROS was detected by MitoSOX^TM^ Red Mitochondrial Superoxide Indicator (Invitrogen, Thermo Fisher Scientific, Shanghai, China). After discarding the culture medium, NRVMs were incubated with 5 μM staining solution at 37°C for 10 min, washed three times with PBS, and then observed under a microscope.

### TdT-Mediated dUTP Nick-End Labeling (TUNEL) Assay

The TUNEL detection kit was purchased from Beyotime. Briefly, NRVMs were cultured in 24-well plates. The cells were incubated with PBS containing 0.5% Triton X-100 at room temperature for 5 min. After adding TUNEL detection solution and incubating at 37°C for 60 min, the tablets were sealed with anti-fluorescence quenching solution and observed under a fluorescence microscope.

### Immunofluorescence

The cells were grown in a 24-well plate and fixed with 4% paraformaldehyde for 30 min. After washing with PBS three times, 0.5% Triton X-100 was added to NRVMs and incubated for 15 min. Next, donkey serum was used to seal the cells for 30 min, and the cells were incubated with the primary antibodies in a 4°C refrigerator overnight. After washing with PBS 3 times, the secondary antibodies were incubated at room temperature for 1 h. PBS was rinsed three times successively, and then cell nuclei were stained with DAPI and observed under a fluorescence microscope.

### Magic Red Cathepsin Assay

Neonatal rat ventricular myocytes were seeded into 96-well plates with black walls and a clear bottom and treated with different concentrations of nicotine, Torin1 or NAC. DMSO (50 μL) was added to the CTSL assay to prepare 260 × original solution, which was then diluted with 450 μl aseptic water to 26 × staining solution. After discarding the culture media, the dye solution was added to the cells and then incubated at 37°C for 45 min. After washing with PBS 3 times, the red fluorescence intensity was analyzed using a fluorescence plate reader with the optimal excitation and emission wavelength tandem of 592 nm and 628 nm, respectively.

### Statistical Analyses

Data are presented as the mean ± SEM. The statistical analysis of differences between two groups was assessed with the unpaired *t*-test, and differences among three or more groups were assessed by one-way ANOVA followed by Bonferroni’s test for *post hoc* analysis and multiple comparison tests with Prism Software version 6.0 (GraphPad Software, San Diego, CA, United States). Figures were processed with Adobe Photoshop software (Adobe Systems Inc., San Jose, CA, United States). The mean values were derived from at least three independent experiments. Differences with *P* < 0.05 were considered significant.

## Results

### Nicotine Impairs Parkin/PINK1-Mediated Mitophagic Flux in the Late Stage of Mitophagy

**FIGURE 1:**
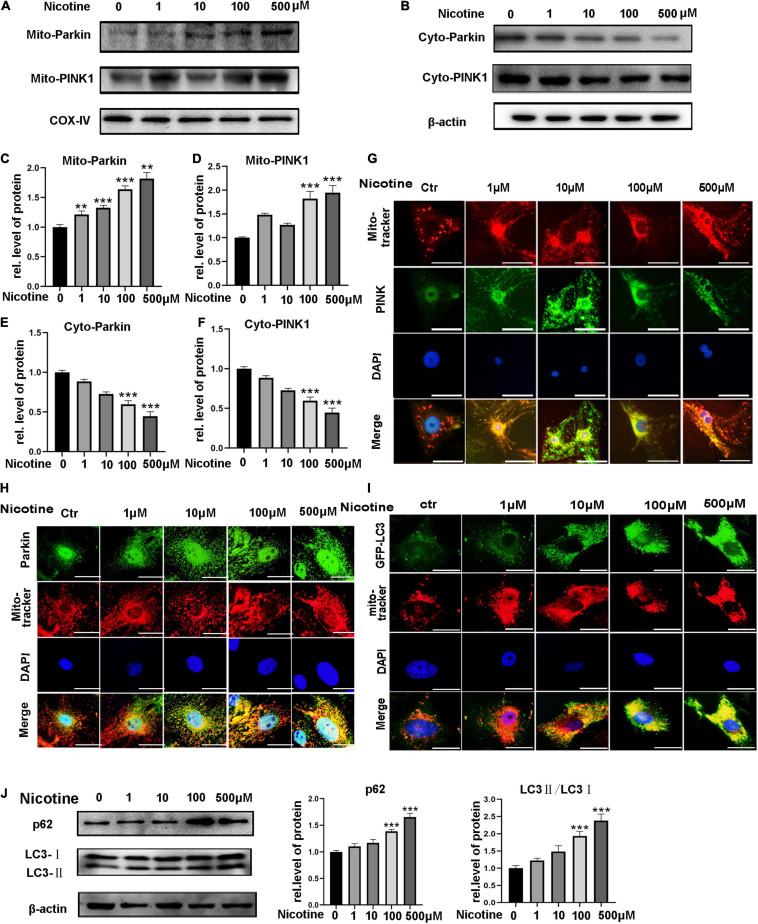
Nicotine impaired mitophagic flux via PINK1/Parkin pathway. **(A,B)** PINK1 and Parkin expression levels were tested by western blotting in mitochondria and cytoplasm. **(C–F)** Image J was used to quantification (***p < 0.001; **p < 0.01, *n* = 3). **(G,H)** Immunofluorescence assay for the distribution of PINK1 and Parkin in cytoplasm and mitochondria **(I).** Co-localization of LC3 and mito-tracker was used to detect mitophagic flux with nicotine treatment for 24 h. Representative of *n* = 3 experiments. (Scale bar = 20 μm) **(J)** Western blotting was used to explore the expression of p62 and LC3 with nicotine treatment for 48h.

To investigate the impacts of nicotine on mitophagy, NRVMs were exposed to different concentrations of nicotine. Western blotting analysis showed that PINK1 and Parkin, two classical molecules of the mitophagy pathway, accumulated in mitochondria but decreased in the cytoplasm after nicotine treatment, indicating that PINK1 and Parkin translocated from the cytoplasm to mitochondria and accumulated in mitochondria (Figures 1A–F). PINK1/Parkin-mediated mitophagy is initiated by the accumulation of PINK1 at the outer membrane of mitochondria, leading to the recruitment of cytoplasmic Parkin to those mitochondria. To investigate further the mitophagy process, the localization of Parkin and Pink1 after nicotine stimulation was examined by immunofluorescence staining. As shown in Figures 1G,H, upon stimulation with nicotine, the expression of PINK1 and Parkin was enhanced in mitochondria. Parkin was translocated to mitochondria from the cytoplasm.

To determine mitophagic flux, we observed the co-localization of GFP-LC3 and mito-tracker. The results showed LC3 marked mitophagosomes accumulated with nicotine treatment. And then, western blotting was used to detect the expression of p62 and LC3. We observed the expression of p62 and LC3-II increased, suggesting mitophagic flux impaired with nicotine treatment (Figures 1I,J).

### Nicotine Stimulation Caused an Imbalance in Mitochondrial Fusion and Fission

**FIGURE 2:**
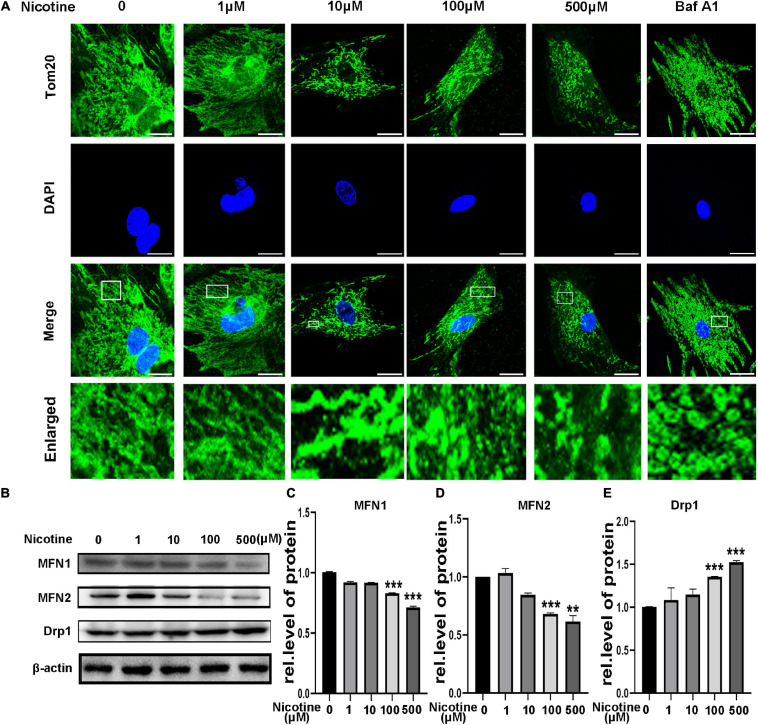
Nicotine damaged mitochondrial morphology and altered dynamics. **(A)** Confocal microscopy was used to observe mitochondrial morphology of NRVMs treated with nicotine. BafA1 (100 nM) was used as positive control. Representative images of NRVMs treated with nicotine or BafA1 for 24 h. Representative of three independent experiments (Scale bar, 10 μm). **(B)** Western blotting was performed to detect mitochondrial morphology and dynamics factors MFN1, MFN2, Drp1 protein levels in NRVMs after treating with nicotine. **(C–E)** Image J was used to quantification (***p < 0.001; **p < 0.01, *n* = 3).

Mitochondrial fusion and fission have been proven to be related to mitophagy (Zhang et al., 2019). However, whether nicotine-induced mitophagic flux impairment contributes to an imbalance in mitochondrial dynamics is unknown. First, NRVMs were treated with bafilomycin A1 (BafA1), an acknowledged autophagy inhibitor. After stimulation with BafA1, mitochondria showed more round, punctate dot-like fragments, similar to nicotine treatment (Figure 2A), indicating that blocked mitophagy may be the reason for mitochondrial dynamics disorder. To determine further the effects on mitochondrial fusion and fission, we determined the expression levels of the mitochondrial fusion biomarkers MFN1 and MFN2 and the mitochondrial fission-related protein Drp1. Western blotting revealed that MFN1 and MFN2 decreased and the level of Drp1 was elevated following nicotine treatment (Figures 2B–E). Moreover, the transcriptional levels of MFN1 and MFN2 were downregulated, while the transcripts of pro-fission Drp1 were increased (Supplementary Figures 1A–C). Based on the above, we demonstrated that nicotine increased mitochondrial fission but decreased mitochondrial fusion via mitophagy impairment.

### Nicotine Induced Mitophagic Flux Impairment by Decreasing CTSL Activity, Activating mTOR, and the ROS-Mediated p38/JNK Pathway

**FIGURE 3:**
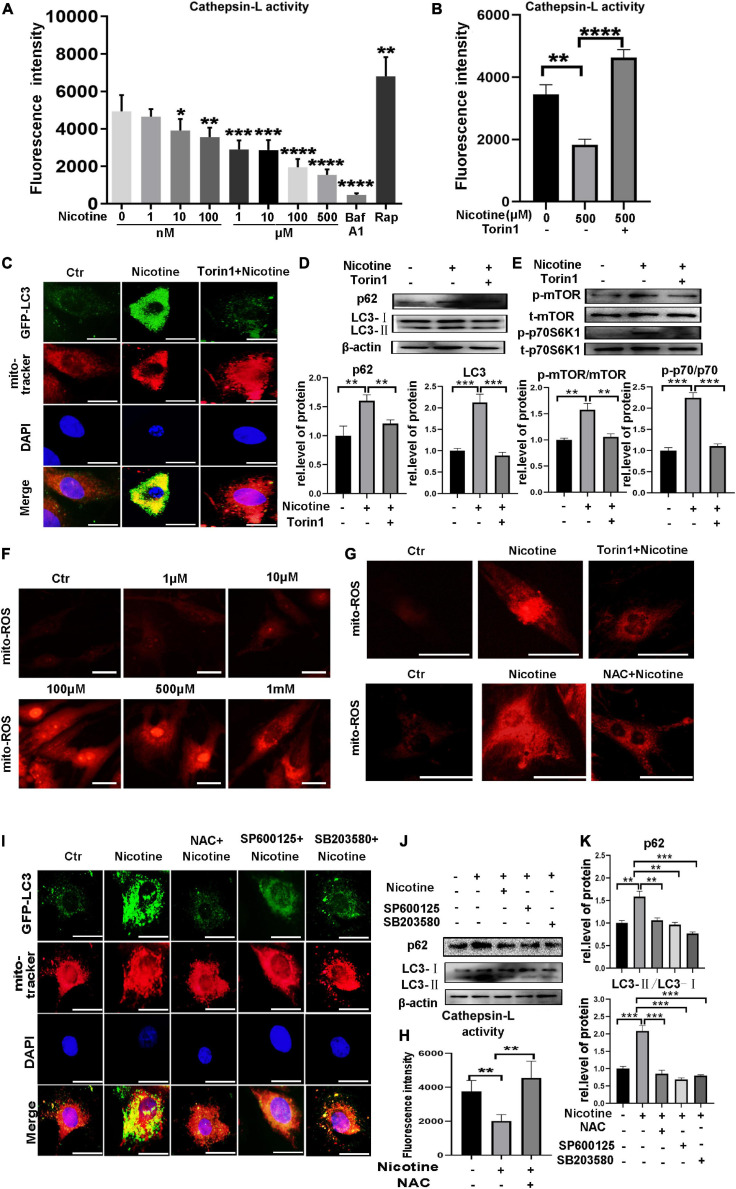
Nicotine caused mitophagy impairment through decreased the CTSL enzyme activity via ROS mediated p38/JNK pathway. **(A)** Nicotine decreased the activity of CTSL. **(B)** Torin1 alleviated the activity of CTSL damaged by nicotine. **(C)** Immunofluorescence co-localization analysis was used to detect the effect of Torin1 after nicotine treatment. Representative of *n* = 3 experiments. (Scale bar = 20 μm). **(D)** Western blotting was used to detect the expression of p62 and LC3. **(E)** Western blotting was used to detect the activation of mTOR pathway**. (F,G)** Mito-ROS was detected by mito-SOX (Scale bar = 20μm). **(H)** CTSL activity was restored with NAC pretreatment **(I)** Mitophagic flux impairment was rescued by NAC, SP100625 and SB203580. Representative of *n* = 3 experiments. (Scale bar = 20 μm). **(J)** Western blotting was used to detect the expression of p62 and LC3. **(K)** Image J was used to quantification (****p < 0.0001. ***p < 0.001. **p < 0.01. *p < 0.05, *n* = 3).

Our previous study clarified that nicotine can induce autophagy impairment by inhibiting CTSB enzyme activity. Here, we show that nicotine stimulation could cause a decline in the enzyme activity of CTSL, which is also a member of the lysosomal protease family, playing an important role in the late stage of autophagy. As shown in Figure 3A, the activity of CTSL was inhibited with nicotine stimulation. Interestingly, Torin1, which is an inhibitor of mTOR, can be used to activate CTSL activity (Zhou et al., 2013). Thus, we further detected the activity of CTSL with Torin1 pretreatment. Indeed, as shown in Figure 3B, Torin1 reversed the activity of CTSL repressed by nicotine. In addition, Torin1 pretreatment reduced LC3 marked mitophagosomes and decreased the expression of p62, demonstrating the impaired mitophagic flux was rescued by Torin1 (Figures 3C,D). It has been reported that excessive activation of mTOR pathway can lead to impaired mitophagy (Zhang et al., 2021). We also detected the phosphorylation level of mTOR and p70S6K1 with nicotine treatment. The results showed nicotine increased the phosphorylation level of mTOR and its downstream factor p70S6K1. Torin1 pretreatment could alleviate the effects of nicotine on the over-activation of mTOR (Figure 3E).

It has been reported that mitochondrial reactive oxygen species (mito-ROS) play a crucial role in mitophagy impairment (Joselin et al., 2012). The results demonstrated that mito-ROS exhibited pathological accumulation under nicotine stimulation (Figure 3F). Interestingly, ROS production was downregulated by Torin1 pretreatment. NAC, an ROS scavenger, was used as a positive control (Figure 3G). Importantly, the activity of CTSL was restored, and mitophagy impairment was rescued after NAC pretreatment (Figure 3H). P38 and JNK are two key factors downstream of ROS and have also been proven to be involved in the regulation of mitophagy (Ma et al., 2019). Our previous work clarified that p38/JNK activation involved in nicotine caused macroautophagic flux impairment (Wang et al., 2020). Here, we also detected mitophagic flux after pretreatment with SP600125 and the p38 MAPK inhibitor SB203580 (Figures 3I,J). The results indicated that the ROS-mediated p38/JNK signaling pathway also impacted PINK1/Parkin-mediated mitophagic flux. In summary, these results demonstrated that nicotine could inhibit mitophagic flux by decreasing the enzyme activity of CTSL and activating ROS-dependent p38/JNK signaling pathways. Torin1 could alleviate damaged mitophagy by restoring the activity of CTSL, inhibiting the activation of mTOR pathway and alleviating ROS accumulation, mitigating nicotine-induced mitophagic flux impairment.

### Hindered Mitophagic Flux via Decreased CTSL Activity and Activation of the ROS-Mediated p38/JNK Pathway Contributed to Nicotine-Induced Mitochondrial Fusion and Fission Imbalance

**FIGURE 4:**
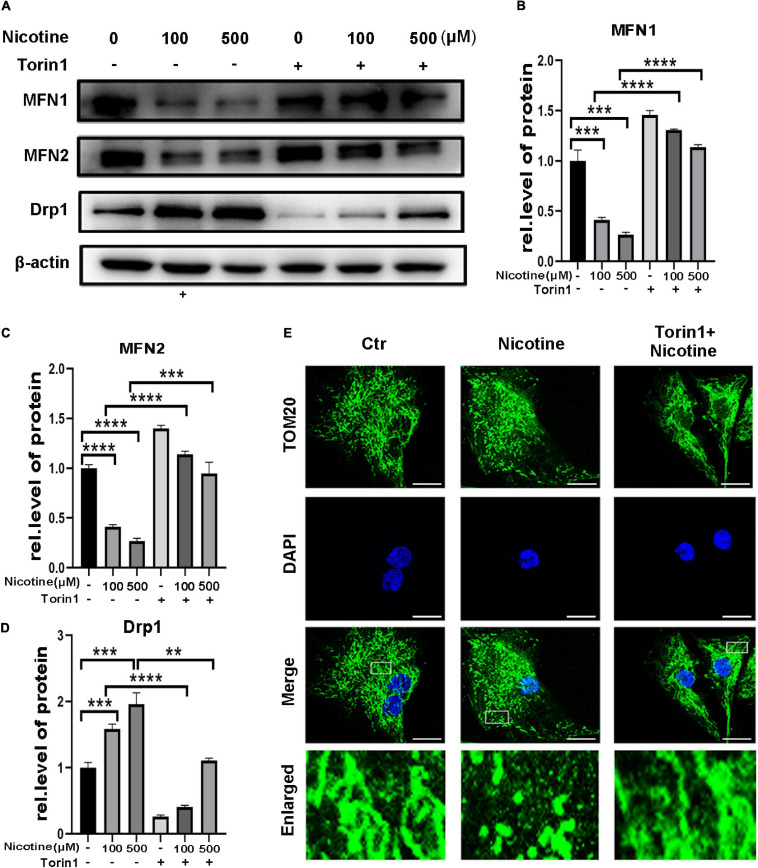
Torin1 relieved mitochondrial dynamics disorder induced by nicotine. **(A)** Western blotting was performed to detect mitochondrial dynamics factors with pretreatment of Torin1. **(B–D)** Protein levels were quantified by Image J. (****p < 0.0001; ***p < 0.001; **p < 0.01, *n* = 3). **(E)** Confocal microscopy was used to detect the mitochondrial morphology with the treatments of Torin1 (Scale bar = 10 μm).

To determine further the effects of hindered mitophagy flux on mitochondrial dynamics. NRVMs were pretreated with Torin1, which could restore mitophagy flux inhibited by nicotine. As shown in Figure 4, Torin1 alleviated nicotine-induced disorder of mitochondrial dynamics as evidenced by elevation of the protein levels of the mitochondrial fusion factors MFN1 and MFN2 and decreased levels of Drp1 in NRVMs (Figures 4A–D). Moreover, the transcriptional levels of pro-fusion factors were upregulated in response to Torin1 pretreatment (Supplementary Figures 1D,E). In contrast, mitochondrial fission factor was downregulated by Torin1 (Supplementary Figure 1F). Similarly, confocal microscopy revealed that Torin1 ameliorated mitochondrial fragmentation induced by nicotine (Figure 4E). These results demonstrated that the mitochondrial dynamics disorder induced by nicotine could partially be recovered by treatment with Torin1.

**FIGURE 5:**
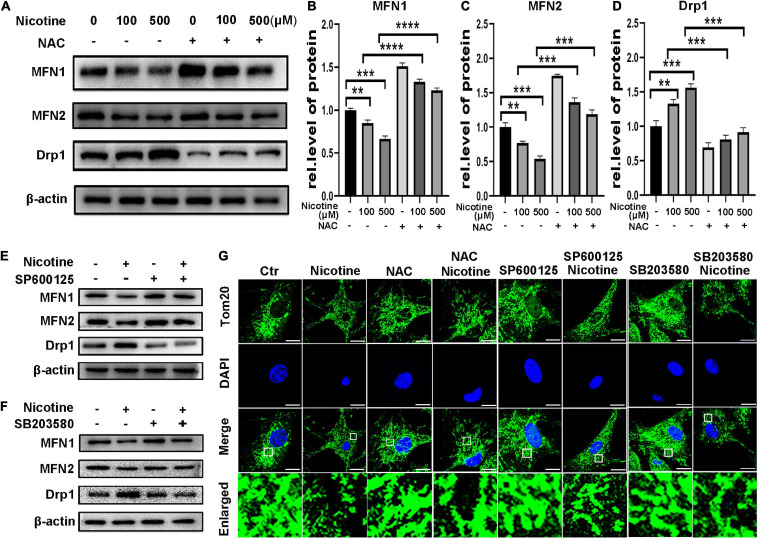
Nicotine induced mitochondrial dynamics disorder through ROS mediated p38/JNK pathway. **(A)** Protein levels of mitochondrial dynamics factors with NAC pretreatment. **(B–D)** Results were analyzed by Image J. **(E,F)** Pre-treatment of SP600125 and SB203580 alleviate mitochondrial dynamics disorder, which shown by western blotting. **(G)** Mitochondrial morphology was observed by Laser confocal microscopy imaging system with pretreatment of NAC, SP600125 and SB203580 in NRVMs (Scale bar = 10 μm). (****p < 0.0001; ***p < 0.001; **p < 0.01, *n* = 3).

To determine further whether ROS-mediated MAPK signaling is involved in the regulation of nicotine-mediated changes in mitochondrial dynamics, NRVMs were exposed to nicotine by pretreatment with NAC, the JNK inhibitor SP600125 and the p38 MAPK inhibitor SB203580. Pretreatment with the NAC and JNK inhibitor SP600125 and the p38 MAPK inhibitor SB203580 also alleviated the effects of nicotine and Torin1. As shown in Figure 5, MFN1 and MFN2 protein levels were increased, while Drp1 was decreased after NAC treatment (Figures 5A–D). Consistent with these results, qPCR analysis illustrated that NAC treatment inhibited nicotine-induced Drp1 upregulation and increased the levels of mitochondrial fusion factors (Supplementary Figures 1G–I). Western blotting also revealed that mitochondrial dynamics disorder was rescued by the JNK inhibitor SP600125 and p38 MAPK inhibitor SB203580 (Figures 5E,F). Immunofluorescence results showed that pretreatment with NAC and two inhibitors protected mitochondria from becoming punctate and fragmented spots (Figure 5G), indicating that mitophagy impairment may be the reason for mitochondrial dynamics disorder.

### Nicotine Induced Cardiomyocyte Apoptosis via ROS Accumulation and Mitochondrial Fission *in vitro*

**FIGURE 6:**
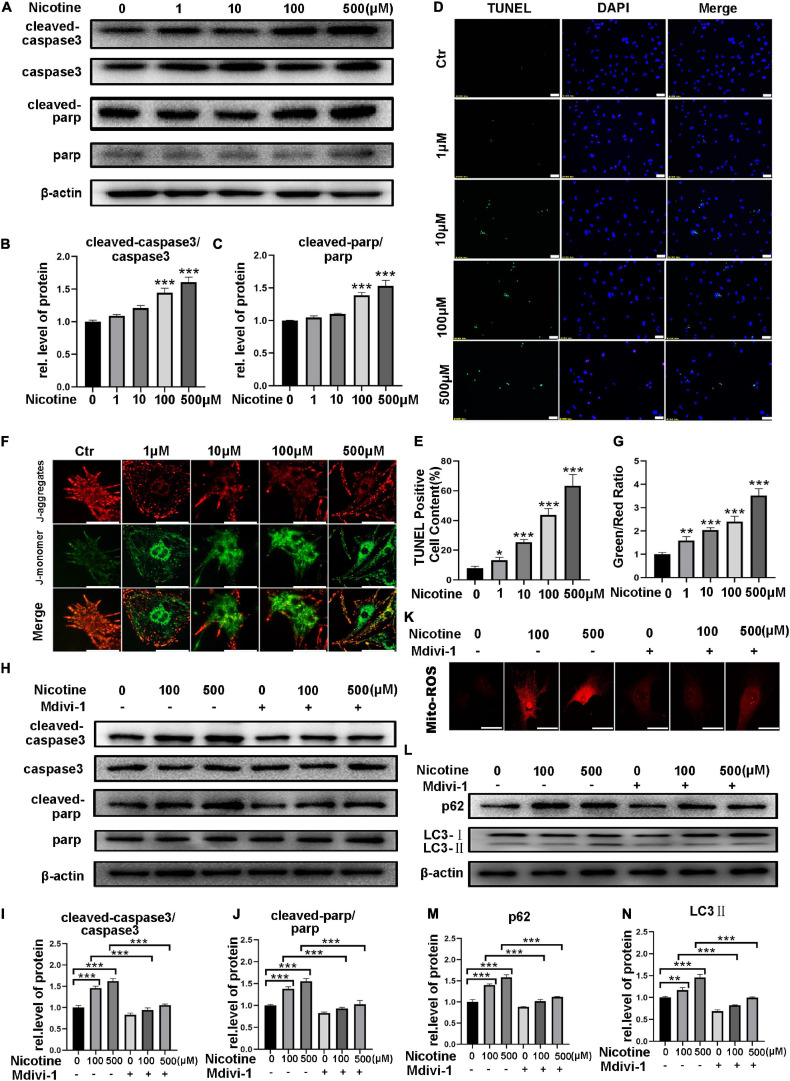
Nicotine caused cardiomyocyte apoptosis, which attenuated by mdivi-1. **(A)** Western blotting was performed to detect apoptosis markers cleaved-caspase3, caspase3, cleaved-PARP, PARP protein levels of NRVMs after treating with nicotine. **(B,C)** Image J was used to quantification (***p < 0.001, *n* = 3)**. (D–E)** TUNEL assay of apoptotic cardiomyocytes induced by nicotine (Scale bar = 50 μm). **(F,G)** JC-1 staining was used to demonstrate the variety of mitochondrial membrane potential (Scale bar = 20 μm). **(H–J)** Western blotting analysis of apoptosis related protein treated by mdivi-1. **(K)** Mdivi-1 decreased the generation of mito-ROS (Scalebar = 20 μm). **(L)** Western blot was performed to determine the autophagy marker LC3-II and its specific substrate p62 expression. **(M,N)** Image J was used to quantification (***p < 0.001; **p < 0.01; *p < 0.05, *n* = 3).

Previous studies have shown that Drp1 impacts cell sensitivity to cell death and further relates the mitochondrial fission mechanism to apoptosis (Suen et al., 2008). Silencing of MFN1 or MFN2 also results in mitochondrial fragmentation and an increase in sensitivity to apoptotic stimuli (Sugioka et al., 2004). Thus, we further explored the effects of nicotine on cardiomyocyte apoptosis. NRVMs were exposed to different concentrations of nicotine for 48 h, and cleaved caspase-3 and cleaved PARP were significantly increased with nicotine treatment (Figures 6A–C). In line with these results, a TdT-mediated dUTP nick-end labeling (TUNEL) staining assay showed that positive spots significantly increased in NRVMs with nicotine stimulation in a dose-dependent manner (Figures 6D–E). Moreover, mitochondrial membrane potential, which is a landmark event in the early stage of apoptosis, was tested by JC-1 staining. As presented in Figures 6F,G, the mitochondrial membrane potential decreased significantly after nicotine treatment.

To determine whether mitochondrial fission and mitophagy contribute to apoptosis, NRVMs were treated with mdivi-1, an inhibitor of Drp1. As shown in Figure 6H, cleaved PARP and cleaved caspase-3 decreased after pretreatment with midiv-1 compared with the nicotine treatment group (Figures 6H–J). Accordingly, the nicotine group showed more TUNEL-positive puncta and more ROS without pretreatment with mdivi-1 (Figure 6K and Supplementary Figure 2A). Furthermore, pretreatment with mdivi-1 alleviated mitophagy impairment induced by nicotine, manifesting as a significant decrease in LC3-II and p62 in NRVMs, suggesting that mitochondrial fission could be involved in the regulation of autophagy (Figures 6L–N). Adenovirus transfection of Fis1-RFP-GFP also revealed that mitophagy impairment was rescued by mdivi-1 (Supplementary Figure 2B).

**FIGURE 7:**
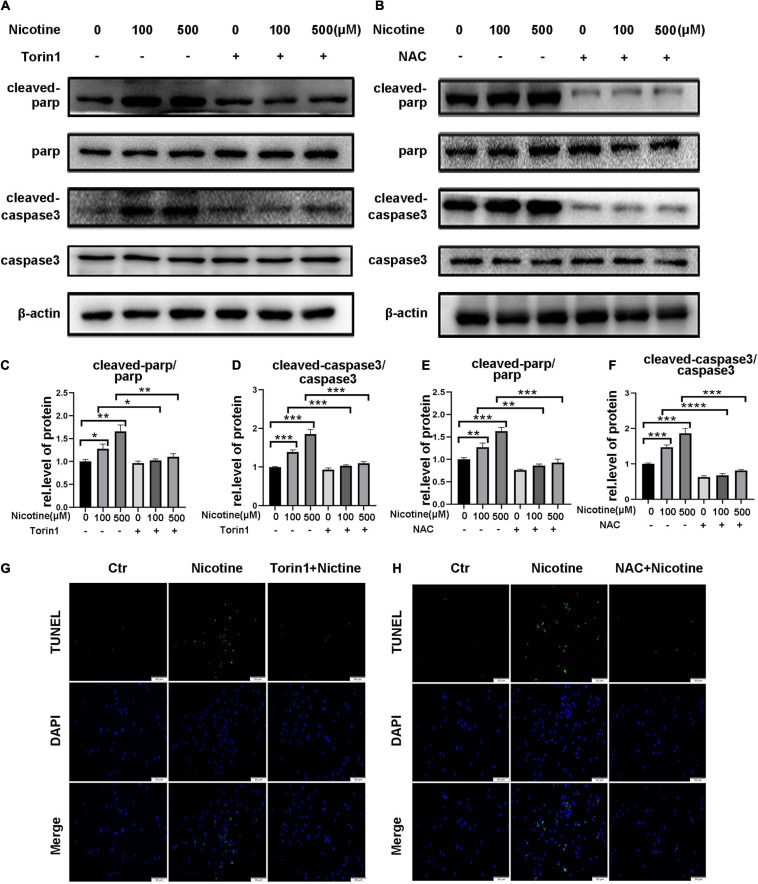
Torin1 and NAC reduced the rate of myocardial apoptosis affected by nicotine. **(A–F)** Western blotting analysis and its quantification of apoptosis related protein. (****p < 0.0001; ***p < 0.001; **p < 0.01; *p < 0.05, n = 3). **(G,H)** TUNEL staining of NRVMs pretreated with Torin1 or NAC followed by nicotine exposure.

Torin1 and NAC, which alleviated nicotine-induced mitophagic flux impairment and mitochondrial dynamics disorder, also prevented apoptosis in NRVMs as demonstrated by decreased expression of cleaved PARP and cleaved caspase 3 compared with nicotine injury (Figures 7A–F) and reduced number of TUNEL-positive cells compared with the nicotine group (Figures 7G,H). Taken together, excessive mitochondrial fission contributes to apoptosis in NRVMs and preventing mitophagic flux impairment may be a feasible strategy for preventing mitochondrial dysfunction and promoting cell survival. In the present study, our results demonstrated that nicotine treatment results in an imbalance of mitochondrial dynamics and apoptosis by hindering PINK1/Parkin-mediated mitophagic flux by decreasing CTSL activity and the ROS-mediated p38/JNK pathway, while Torin1 attenuates nicotine injury by rescuing mitophagy impairment.

## Discussion

The heart is a highly energy-consuming organ, and the normal function of mitochondria plays an important role in the heart (Dorn et al., 2015). Mitochondrial dysfunction is an important contributor to the development and pathogenesis of heart failure. Mitochondrial dynamics play a crucial role in maintaining mitochondrial morphology and functions to meet cardiomyocyte energy requirements. Mitochondrial fission and fusion are involved in the regulation of various biological processes, such as proliferation, mitochondrial redistribution, cell cycle, and apoptosis (van der Bliek et al., 2013). However, whether nicotine impacts mitochondrial dynamics in NRVMs is unclear. Our results showed that nicotine stimulation caused excessive mitochondrial fission and reduced fusion in NRVMs. These results were consistent with Naoya Hirata’s report in human multipotent embryonic carcinoma cells (Hirata et al., 2016).

In addition, mitochondrial dynamics are closely linked to mitochondrial quality control, especially mitophagy (Xian and Liou, 2020). Mitochondrial fragmentation is coupled with mitochondrial membrane potential repression and precedes mitophagosome formation (Kissová et al., 2004; Narendra et al., 2008; Ding et al., 2010). Mitochondrial fusion was reported to promote protective mitophagy (Maneechote et al., 2017; Zhang et al., 2019; Zhou et al., 2018). Ultra-high fused mitochondria caused by Drp1 deletion can still promote the occurrence of mitophagy (Youle and van der Bliek, 2012; Friedman and Nunnari, 2014; Xian et al., 2019) which calls into doubt the mainstream concept that mitochondrial fission is a precondition for mitophagy. Thus, more research is needed to explore the relationship between mitophagy and mitochondrial dynamics. Whether mitophagic flux impairment is related to mitochondrial dynamics in NRVMs is still unknown. We demonstrated that nicotine-induced mitochondrial fission increased but decreased mitochondrial fusion via PINK1/Parkin-mediated mitophagy impairment. Furthermore, our results also clarified that nicotine repressed CTSL activity, while Torin1 rescued it by restoring mitophagic flux impairment. ROS-dependent p38/JNK activation plays an important role in nicotine-induced autophagic flux impairment, as demonstrated in our previous study (Wang et al., 2020). Thus, we further detected the relationship between mitochondrial dynamics and mitophagy impairment. Our study found that nicotine damaged mitophagic flux by repressing CTSL activity and activating the ROS-mediated p38/JNK pathway, causing mitochondrial dynamics disorder. Pretreatment with the ROS scavenger NAC, SP600125 and the p38 MAPK inhibitor SB203580 reduced the cardiotoxicity of nicotine. These results may provide a new potential explanation for mitochondrial-associated mitophagic flux impairment under some pathological conditions.

A previous study reported that mitochondrial fission was pivotal in apoptosis caused by different stimuli (Karbowski et al., 2002; Xie et al., 2018). Our results showed that nicotine increased mitochondrial fission and that apoptosis also occurred. Pretreatment with mdivi-1, which is a mitochondrial fission inhibitor, alleviated nicotine-induced apoptosis, suggesting that nicotine-induced mitochondrial fission contributed to apoptosis. NAC, an ROS scavenger that ameliorates nicotine-induced mitophagy flux impairment and mitochondrial dynamics imbalance, rescues nicotine-triggered apoptosis. Since NRVMs cannot be renewed, maintaining the fitness of the network among mitophagy, mitochondrial dynamics, and apoptosis is crucial. Our study indicated that excessive mitochondrial fission and ROS accumulation under stimulation with nicotine eventually triggered apoptosis of NRVMs. Silencing of Mfn1 or Mfn2 was proven to increase the sensitivity of cells to apoptosis (Sugioka et al., 2004; Suen et al., 2008). Thus, the equilibrium of mitochondrial dynamics may be critical in the regulation of apoptosis, which still needs further investigation.

Taken together, our study showed that nicotine induced mitochondrial dynamics disorder by blocking mitophagic flux by repressing CTSL activity and activating ROS-mediated p38/JNK pathways, while Torin1 rescued NRVMs by restoring CTSL activity and damaged mitophagic flux. These results may reveal a close link between mitophagy and mitochondrial dynamics, which also provides new evidence of the effects of nicotine on mitochondrial dynamics in NRVMs.

## Data Availability Statement

The raw data supporting the conclusions of this article will be made available by the authors, without undue reservation.

## Ethics Statement

The animal care and experimental procedures involved in this study were reviewed and approved by Ethics Committee of Jinan Central Hospital (approval code: AF/SC-07/02.0).

## Author Contributions

T-TM and YL designed the study. T-TM, WW, and S-YW performed the experiments. F-LM, J-MC, and H-HW analyzed data. T-TM wrote the manuscript. YZ, G-XW, and M-XZ revised the article. YL and G-HS helped to draft the manuscript. All authors read and approved the final manuscript.

## Conflict of Interest

The authors declare that the research was conducted in the absence of any commercial or financial relationships that could be construed as a potential conflict of interest.

## Supplementary Material

Supplementary Figure 1 **(A–I)** MFN1, MFN2, and Drp 1 level were determined by qPCR (^****^*p* < 0.0001; ^∗∗∗^*p* < 0.001; ^∗∗^*p* < 0.01; and ^∗^*p* < 0.05, *n* = 3).

Supplementary Figure 2 **(A)** TUNEL assay of NRVMs with mdivi-1 pretreatment (Scale bar=50 μm). **(B)** RFP-GFP-Fis1 transfection were used to test the effect of Mdivi-1(Scale bar = 20 μm).
