# Supplementary figures and images for: Nicotine Causes Mitochondrial Dynamics Imbalance and Apoptosis Through ROS Mediated Mitophagy Impairment in Cardiomyocytes
Source: Front Physiol. 2021 Jun 10;12:650055. doi: 10.3389/fphys.2021.650055 (PMC8222989; doi:10.3389/fphys.2021.650055)

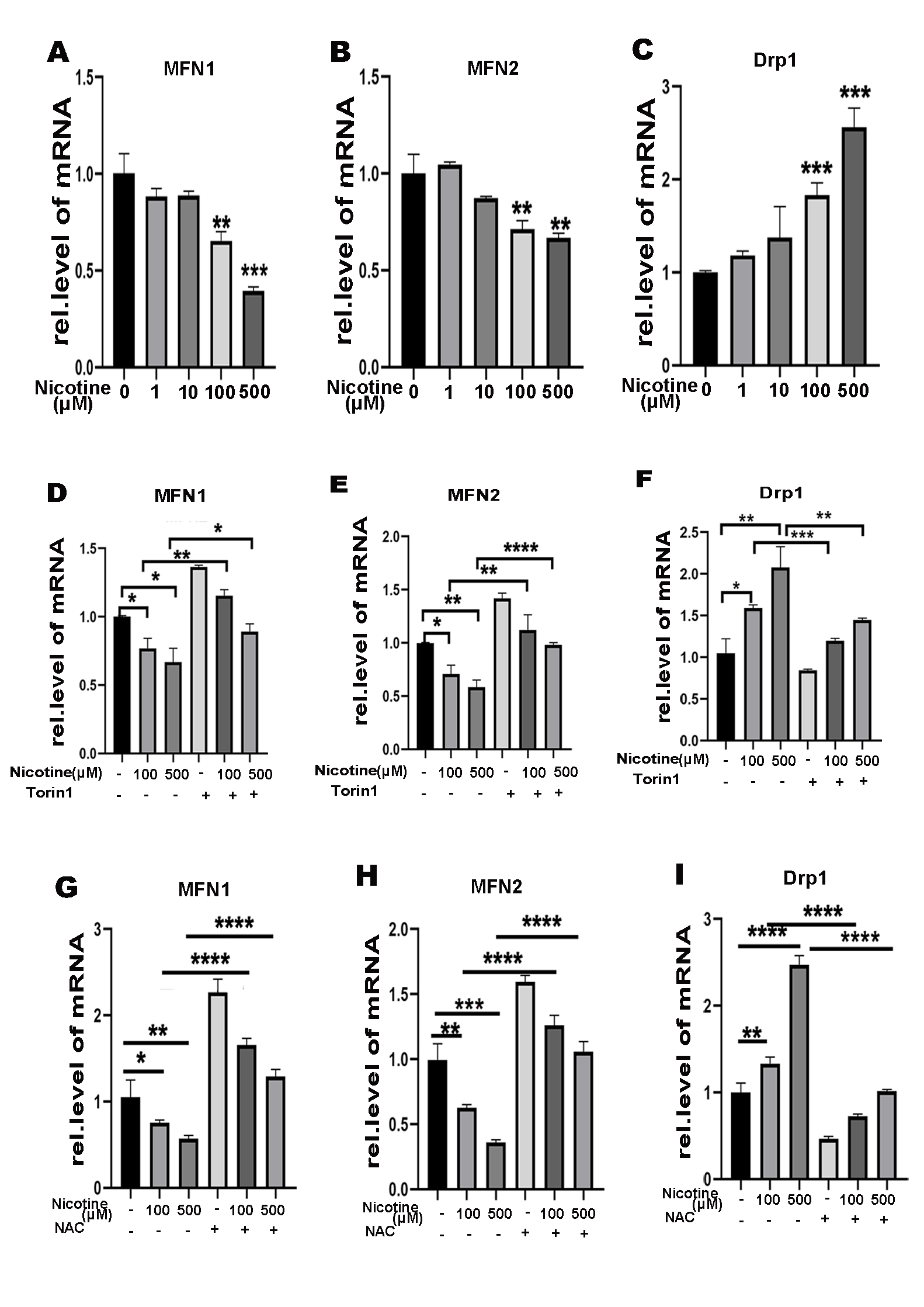

Supplement: Supplementary Figure 1 — (A–I) MFN1, MFN2, and Drp 1 level were determined by qPCR (****p < 0.0001; ∗∗∗p < 0.001; ∗∗p < 0.01; and ∗p < 0.05, n = 3). [file Image_1.TIF]

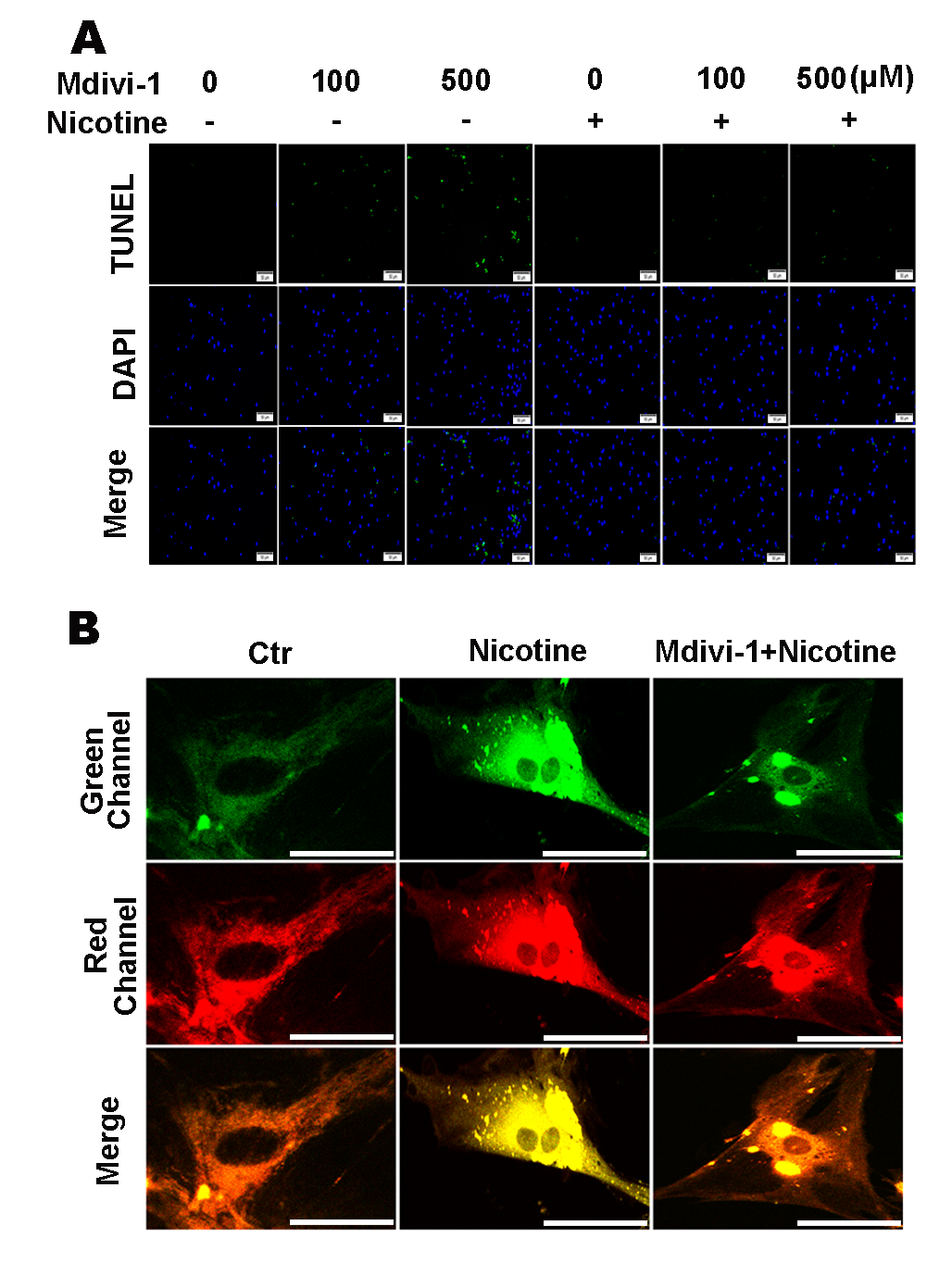

Supplement: Supplementary Figure 2 — (A) TUNEL assay of NRVMs with mdivi-1 pretreatment (Scale bar=50 μm). (B) RFP-GFP-Fis1 transfection were used to test the effect of Mdivi-1(Scale bar = 20 μm). [file Image_2.TIF]
